# The American Academy of Dental Surgery

**Published:** 1877-05

**Authors:** 


### AMERICAN JOURNAL

OF

### DENTAL SCIENCE.

Vol. XI. THIRD SERIES—MAY, 1877. No. 1.

### ARTICLE I.

### *The American Academy of Dental Surgery.*

[Reported for the American Journal of Dental Science.]

The regular meeting of this organization occurred on Tuesday evening, March 27th. The meeting was called to order by the President, Dr. Geo. H. Perine, and minutes of the last meeting were read and approved. The Chairman of the Executive Committee recommended several applicants for fellowship, who were unanimously elected.

The Treasurer's, Secretary and Curator's reports, were read, adopted, and ordered to be placed on file. Donations received and recorded.

After miscellaneous business had been completed, Dr. Brush reported an operation performed by J. F. Miner, M. D., removal by Galvano Cautery, Malignant Diseased Ton sil, which seriously interfered with both respiration and deglutition, and was also causing considerable annoyance from the amount of pain it produced. The patient, a man of about sixty-three years, presented an anxious, cachectic appearance, and though of an apparently sturdy constitution, he now looked ill and worn out. An examination of the throat revealed a tumor springing from the seat of the right tonsil and almost filled the entire throat; it was modulated in appearance, soft, and bled on slight manipulation. The discharge from the surface was not very profuse, but had a very disagreeable odor, the glands in the neck, on the corresponding side were enlarged. The tumor in the throat had been of somewhat rapid growth, the exact duration of which was not however at the time noted. No family history of cancer was elicted. The diagnosis made, was encephaloid disease of the tonsil, and after carefully weighing the dangers of the operation, its removal was advised, not with the idea of a permanent cure, but for the purpose of affording temporary relief, which was all that was expected. An attempt was made to pass a wire loop of the galvano-cautery apparatus about the base of the tumor, but owing to the shape of the growth and its situation, this was found impossible. The platinum knife was next heated to a white heat and the larger portion of the tumor shaved off with it. It was found impossible without injury to the surrounding tissues to remove the entire tumor in this manner, and recourse was had to a pair of long curved scissors, by which all remaining fragments of the tumor was removed; the hemorrhage from the cut surface, which was quite free, was easily controlled by the dome cauterizer heated by the battery. The surface left after the removal of the tumor and the application of the cautery, had a smooth even feeling, and was evidently in good condition for whatever reparative attempt nature might make. The enlarged glands in the neck were also removed.

When the patient again presented himself for examination and advice, the tumor in the throat had not again made its appearance, but the site of the original growth had an healthy look. In the neck the glands had enlarged, and at the site of those removed, a tumor had attained the size of an English walnut. No operative procedure was advised, the object of the original operation having been attained viz;—removal of the obstruction to deglutition and respiration.

Malignant disease of the tonsil is not of a very frequent occurrence. There is nothing however in this case which warrants a report of its details, except the facility with which the operation was made. The risk attending an operation of this character, and upon which some surgeons have laid considerable stress, have heretofore been in the danger of uncontrollable hemorrhage, but surgeons now have at their command an apparatus by which the actual cautery can be applied with ease, precision, and at a moment's notice, and by the employment of the galvano-cautery, the danger surrounding this operation is in a great measure removed.

Dr. Mercer said he had great confidence in the battery for operations in the oral cavity. He had witnessed an operation made by Dr. Perine, the removal of a tumor from the mouth of a patient, with the galvano-cautery, and he would like to here from that gentleman on the subject.

Dr. Perine said he had invited the attention of the dental profession to this subject some time since, and he was pleased to know that they were becoming interested. He had found the cautery battery very useful in dental practice; indeed no agent that human skill shall devise can ever equal electricity; there are so many different ways of applying it, not only in surgery, but in medical practice. Our ignorance of its workings is no bar to our progress in the knowledge of its effects. In employing electricity, just as in the use of medicaments, generally the time required to complete a cure must bear some proportion to the duration of the malady. In affections of Neuralgia, which so often disheartens both the prescriber and the patient, he had found it would yield to the application of electricity. He could cite Neuralgical cases cured with the battery, where medication had failed. Dr. Perine then exibited the working of a galvano-cautery battery which he had made for dental purposes; it seems to possess all the advantages of the larger batteries, and one other which they do not, namely portability, occupying no more space than an ordinary amputating case, and which will continue a white heat about thirty minutes. Dr. Perry said he had used eletricity with much satisfaction in his practice in cases of neuralgia. After some further remarks upon the subject, Dr. Roberts presented the following interesting cases treated by Clinton Wagner, M. D., New York.

Mrs. S. P., aged 41, a native of Ireland, sent to me by Dr. de Bremon, was admitted to the Metropolitan Throat Hospital, May 13. She stated that she first observed a small swelling under her tongue about 15 years ago; it gave rise to no inconvenience until a year ago, when it began to increase. An examination disclosed a large oval mass, or tumor, extending from beyond the lower teeth in front, to the base of the tongue posteriorly, which organ was forced upward and backwards, deglutition and speech were greatly interfered with, and for weeks she had not been able to take solid food.

The tumor was more solid than a ranula, but when pressed between the fingers, imparted the sensation of an encysted mass. An incision was made upon the left side about an inch in length, from which gushed out about three and a-half ounces of a thick, yellowish, and extremely offensive matter.

A portion of the cyst was removed, a large tent inserted, and injections of tinct. iodine made daily to excite adhesive inflammation. In ten days the cavity had closed, and the woman was discharged from the hospital cured.

Microscopically examined by Dr. Rudolf Tauszky, the contents of the cyst were found to consist of "colorless, transparent, rhomboidal plates, deposited in layers, in which the outlines of the subjacent crystals showed very distinctly through the substance of those placed above—characteristic of the crystals of cholesterin, similar to those found in the sebaceous matter of the skin."

Chemically examined, it responded to Dalton's test for cholesterin.

W. Fairlike Clarke describes a tumor somewhat similar to this case, which is found between the tongue and jaw, and which becomes prominent at the upper part of the neck, and to which the term, ranula, is improperly applied; the cure is difficult and tedious, and it is analagous to the sebaceous tumors so frequently met with in the skin.

Mr. F., aet. 39, of Baltimore, consulted me at the suggestion of Dr. Van Bibber of that city. He gave the following history: Five years ago, he first observed the trouble in his nose; it began with an acid discharge; sense of stuffness, fullness or closure of the nostril, the voice assuming a nasal tone. At the time of consulting me, these symptoms had greatly increased, and the discharge was so excessive that almost constant recourse to the handkerchief was necessary.

An examination revealed a large, rounded mass, of a bright red color, soft and yielding to the touch, filling the entire cavity of the right nostril, and distinguishable externally by a well defined prominence. The rhinoscopic examination showed that the posterior nares were not involved. The tumor was sessile, and attached principally to the cartilaginous septum, and to a slight extent to the vomer.

Removal by means of the galvano cautery was considered, but owing to the broad base, and the difficulty of successfully enclosing the whole mass in the loop, the plan was abandoned in favor of the knife. I then introduced and forced the index finger of my left hand along the floor of the nose and under the tumor as far as possible, using it as a guide for the knife, no difficulty was experienced in separating the mass from its attachments.

During the operation very little blood was lost, but a few minutes later, after he had been conveyed to bed and reac tion from the effects of the ether, had begun, frightful hemorrhage took place, anteriorly and posteriorly; syncope ensued before it could be arrested, which was finally done, however, by means of the tampon and Monsell's solution of iron; during the night he vomited several large basins of blood, each effort being followed by fainting, so great was the prostration from the loss of blood.

Under the influence of a generous diet, and a liberal allowance of Burgundy, he recovered sufficiently in a few weeks to return to his home in Baltimore. Eight months have elapsed since the operation, and there has been no return of the tumor; his voice has lost its nasal twang, and with relief from the local annoyance his general health has greatly improved.

Prof. J. W. S. Arnold, who made a microscopical examination of the tumor, described it as a "vascular myxoma, containing more round than process cells."

The nose is a favorite seat of the myxomas, but the vascular variety, particularly of the size of the one just described, is extremely rare; smaller I have frequently met with upon the alae and septum; one or two applications of the London paste, or the ferri perchlor two drachms, to aquae one ounce, is sufficient to destroy them without causing pain or annoyance to the patient. A tumor of this nature thoroughly removed by knife or caustic is not apt to recur.

I had valuable assistance during the operation from Dr. Van Bibber, of Baltimore, and Drs. Asch, McBurney and Desvernine of this city.

An animated discussion followed the presentation of the cases, which was participated in by Fellows present. When meeting adjourned.

